# Supplementary material for: Content-rich biological network constructed by mining PubMed abstracts
Source: BMC Bioinformatics. 2004 Oct 8;5:147. doi: 10.1186/1471-2105-5-147 (PMC528731; doi:10.1186/1471-2105-5-147)
Supplement: Additional File 2 — The original results of the above study (non-essential files are deleted to keep the file size under the limit set by BMC bioinformatics). [file 1471-2105-5-147-S2.bz2 › chilibotAdditionalFile2/dip50/575ID9204896E10943/html/ANGPT1_TEK.html]

 


 **ANGPT1** and **TEK** 
  
Found 178 abstracts in PubMed, retrieved 50.  
 

 What does Google say? 
 PDF only 
| .edu only 

---

**Interactive relationship** (e.g. stimulation, inhibition, etc)

**Stimulatory relationship**- Ang 1  [ **ANGPT1** ]  activates the endothelial cell specific tyrosine kinase receptor Tie 2  [ **TEK** ] , which in turn leads to enhanced endothelial cell survival and stabilization.  Ref: 12810673 Cancer Res, 2003
- Together, these findings demonstrate that tyrosine residue 1106 on Tie2  [ **TEK** ]  is critical for coupling downstream cell migration signal transduction pathways with Ang1  [ **ANGPT1** ]  stimulation in endothelial cells.  Ref: 12665569 Mol Cell Biol, 2003
- Using a combination of immunohistochemistry, laser capture microdissection and real time quantitative reverse transcription PCR, we showed that tumor derived VEGF significantly up regulated the expression of Ang  [ **ANGPT1** ]  2 in host stroma endothelial cells, resulting in markedly increased Ang  [ **ANGPT1** ]  2 Tie 2  [ **TEK** ]  mRNA copy number ratio in vivo.  Ref: 12810677 Cancer Res, 2003
- Tie2  [ **TEK** ]   **Tek**  is an endothelial cell receptor tyrosine kinase that induces signal transduction pathways involved in cell migration upon angiopoietin 1  [ **ANGPT1** ]  Ang1  [ **ANGPT1** ]  stimulation.  Ref: 12665569 Mol Cell Biol, 2003
**Neutral relationship**- ... ephrinB2 did not alter VEGF induced Flk phosphorylation or Ang1  [ **ANGPT1** ]  induced Tie2  [ **TEK** ]  phosphorylation.  Ref: 12039842 FASEB J, 2002
- Recent studies have implicated the Tie2  [ **TEK** ]  tyrosine kinase receptor and its main ligands angiopoietin 1  [ **ANGPT1** ]  Ang 1  [ **ANGPT1** ]  and angiopoietin 2 Ang  [ **ANGPT1** ]  2 as crucial regulators of mural cell recruitment during angiogenesis.  Ref: 12876214 J Cell SciJ Cell Sci, 2002
- Inhibition of interaction of VEGFR 2 or VEGFR 3 with VEGF D but not of Tie 2  [ **TEK** ]  angiopoietin 1  [ **ANGPT1** ]  interaction with soluble receptors abrogated Akt activation and survival of TEC.  Ref: 12709414 FASEB J< MedlineTA>FASEB J, 2002
- Furthermore, in endothelial cells the interaction was stimulated by the Tie2  [ **TEK** ]  ligand angiopoietin 1  [ **ANGPT1** ] .  Ref: 12609966 Circ Res, 2003
- These data provide comprehensive evidence that VEGFR 1 and 2, and angiopoietin 1  [ **ANGPT1** ] , angiopoietin 2 and Tie 2  [ **TEK** ]  interactions may be involved in the preparation of endometrium for implantation, remodelling of the maternal vasculature and trophoblast invasion during the peri implantation period in this primate species.  Ref: 12887279 Reproduction, 2003
- The Tie2  [ **TEK** ]  receptor protein showed the same pattern as Ang  [ **ANGPT1** ]  2.  Ref: 12653688 Clin Sci Lond , 2003
- Two of the Angs, Ang 1  [ **ANGPT1** ]  and Ang  [ **ANGPT1** ]  4, activate the Tie2  [ **TEK** ]  receptor.  Ref: 12783598 Expert Opin Investig Drugs, 2003
- Angiopoietins Ang  [ **ANGPT1** ]  have been recently identified as ligands for Tie 2  [ **TEK** ]  receptor and are thought to be important factors in vascular maturation and stability during angiogenesis.  Ref: 12717391 Hepatology, 2003
- The change in capillary density paralleled with VEGF and Ang  [ **ANGPT1** ]  2 expression, but was inversely related to Tie2  [ **TEK** ]  phosphorylation.  Ref: 12649074 Am J Physiol Heart Circ Physiol< MedlineTA>Am J Physiol Heart Circ Physiol, 2003
- These data suggest that it is endothelial induction of Ang  [ **ANGPT1** ]  2 in tumours that regulates vessel stability and supports targeting Tie2  [ **TEK** ]  as an effective novel anti angiogenic therapy in clear cell RCCs.  Ref: 12434420 J Pathol, 2002
- Angiopoietin 1  [ **ANGPT1** ]  and angiopoietin 2 share the same binding domains in the Tie 2  [ **TEK** ]  receptor involving the first Ig like loop and the epidermal growth factor like repeats.  Ref: 12427764 J Biol Chem, 2003
- Ang1  [ **ANGPT1** ]  and Ang2 bind with similar affinity to the endothelial cell tyrosine kinase receptor Tie2  [ **TEK** ] .  Ref: 12138242 Oncology, 2002
- 1 Angiopoietin 1  [ **ANGPT1** ]  Ang1  [ **ANGPT1** ]  is an angiogenic growth factor that binds to the Tie2  [ **TEK** ]  receptor on vascular endothelium, promoting blood vessel maturation and integrity.  Ref: 12770938 Br J Pharmacol, 2003
- Angiopoietin 1  [ **ANGPT1** ]  Ang1  [ **ANGPT1** ]  is a ligand for the endothelial specific receptor tyrosine kinase  [ **TEK** ] , Tie2  [ **TEK** ] , that protects the adult peripheral vasculature from vascular leakage.  Ref: 12150788 Neuroscience, 2002
- Using primary neuronal cultures, we showed that neurons express Ang 1  [ **ANGPT1** ]  and possess the functional angiopoietin receptor Tie 2  [ **TEK** ] , which is phosphorylated in the presence of Ang 1  [ **ANGPT1** ] .  Ref: 12514118 FASEB J, 2003
- Tie 2  [ **TEK** ]  dependent activation of RhoA and Rac1 participates in endothelial cell motility triggered by angiopoietin 1  [ **ANGPT1** ] .  Ref: 12816861 Blood< MedlineTA>Blood, 2003
- A ligand for TIE2  [ **TEK** ] , Ang 1  [ **ANGPT1** ]  promoted the adhesion of sorted primary Lin low CD34 TIE2  [ **TEK** ]  cells to fibronectin FN, and this adhesion may play a critical role in keeping HSCs in an immature status under the stromal cells.  Ref: 12419314 Biochem Biophys Res Commun, 2002
- Angiopoietin 1  [ **ANGPT1** ]  has recently been shown to stabilize EC networks by binding to the EC specific tyrosine kinase receptor Tie 2  [ **TEK** ] .  Ref: 12138403 Semin Oncol, 2002
- It has been proposed that angiopoietins 1 Ang1  [ **ANGPT1** ]  and 2 Ang2 are pro and anti angiogenic owing to their respective agonist and antagonist signaling action through the Tie2  [ **TEK** ]  receptor.  Ref: 12163646 Proc Natl Acad Sci U S A, 2002
- The angiopoietin Ang  [ **ANGPT1** ]  family of growth factors includes four members, all of which bind to the endothelial receptor tyrosine kinase Tie2  [ **TEK** ] .  Ref: 12783598 Expert Opin Investig Drugs, 2003
- Angiopoietin 1  [ **ANGPT1** ]  Ang 1  [ **ANGPT1** ]  and angiopoietin 2 Ang  [ **ANGPT1** ]  2 have been identified as ligands with different effector functions of the vascular assembly and maturation mediating receptor tyrosine kinase Tie 2  [ **TEK** ] .  Ref: 12427764 J Biol Chem, 2003
- ... Ang  [ **ANGPT1** ]  2 and Ang  [ **ANGPT1** ]  3 inhibit Ang 1  [ **ANGPT1** ]  induced Tie2  [ **TEK** ]  phosphorylation.  Ref: 12783598 Expert Opin Investig Drugs, 2003

**Non-interactive relationship** (e.g. studied together, co-existance, homology, etc.)

- Ang 1  [ **ANGPT1** ]  clearly attenuated serum deprivation evoked apoptosis, an effect which required Tie 2  [ **TEK** ]  receptor activation.  Ref: 12074640 Microvasc Res, 2002
- There was a significant correlation between Ang1  [ **ANGPT1** ]  gene expression and Tie2  [ **TEK** ]  gene expression in normal ovary with CL r = 0.619, p = 0.018.  Ref: 12138242 Oncology, 2002
- Angiopoietin 1  [ **ANGPT1** ]  Ang 1  [ **ANGPT1** ] , a newly discovered ligand of the endothelial specific tyrosine kinase receptor Tie 2  [ **TEK** ] , has been found to promote cell survival, vascular maturation, and stabilization.  Ref: 12690034 Circ Res, 2003
- At different stages of tumor growth, the histological aspects were described and sections were immunostained for VEGF, Ang  [ **ANGPT1** ]  2 and their receptors VEGFR 1, VEGFR 2 and Tie 2  [ **TEK** ] .  Ref: 12174896 Anticancer Res< MedlineTA>Anticancer Res, 2003
- 0.001 and a decrease in Tie2  [ **TEK** ]  receptor mRNA p = 0.001 were observed, but no significant difference was observed in Ang 1  [ **ANGPT1** ]  mRNA abundance between normal kidney and RCC p = 0.37.  Ref: 12434420 J Pathol, 2002
- Using this approach in conjunction with a phosphorylation state specific antibody, we identified tyrosine residue 1106 on Tie2  [ **TEK** ]  as an Ang1  [ **ANGPT1** ]  dependent autophosphorylation site that mediates binding and phosphorylation of the downstream of kinase related Dok R docking protein.  Ref: 12665569 Mol Cell Biol, 2003
- Using RT PCR and immunohistochemistry we demonstrate that testicular vascular endothelial growth factor A VEGF A, ang 1  [ **ANGPT1** ] , ang  [ **ANGPT1** ]  2 and the tie 2  [ **TEK** ]  angiopoietin receptor are expressed in the testis.  Ref: 12773423 Biol Reprod< MedlineTA>Biol Reprod, 2003
- P selectin expression was increased in glomerular capillaries 14 days after disease induction, and capillary loss, as assessed by immunohistochemistry for platelet endothelial cell adhesion molecule, vascular endothelial growth factor VEGF receptor 2 and the angiopoietin Ang  [ **ANGPT1** ]  receptor Tie 2  [ **TEK** ] , was recorded at 14 and 21 days in glomeruli affected by proliferative crescents and or sclerosis.  Ref: 12028448 Kidney Int, 2002
- The level of Ang 1  [ **ANGPT1** ]  and Tie 2  [ **TEK** ]  mRNAs, but not that of Ang  [ **ANGPT1** ]  2 mRNA gradually decreased during a 14 d incubation of cord blood CD34 cells in a liquid culture.  Ref: 12605658 Eur J Haematol, 2003
- To test this hypothesis we measured capillary density, and the expressions of VEGF, angiopoietin 1  [ **ANGPT1** ]  and 2 Ang 1  [ **ANGPT1** ]  and Ang  [ **ANGPT1** ]  2, respectively and the Tie 2  [ **TEK** ]  receptor and its phosphorylation state pY Tie2  [ **TEK** ]  during repetitive episodes of myocardial ischemia in chronically instrumented canines.  Ref: 12649074 Am J Physiol Heart Circ Physiol< MedlineTA>Am J Physiol Heart Circ Physiol, 2003
- While Angiopoietin 1  [ **ANGPT1** ]  obligately activates its Tie2  [ **TEK** ]  receptor, Angiopoietin 2 can activate Tie2  [ **TEK** ]  on some cells, while it blocks Tie2  [ **TEK** ]  activation on others.  Ref: 12361603 Dev Cell, 2002
- Tie 2  [ **TEK** ]  messenger RNA, the receptor of Ang 1  [ **ANGPT1** ]  and Ang  [ **ANGPT1** ]  2, was detected at the same level in FNH as in normal liver.  Ref: 12612904 Gastroenterology, 2003
- Enzyme linked immunosorbent assay based competition assays and co immunoprecipitation experiments analyzing the binding of Ang 1  [ **ANGPT1** ]  and Ang  [ **ANGPT1** ]  2 to truncation mutants of the extracellular domain of Tie 2  [ **TEK** ]  showed that the first Ig like loop of Tie 2  [ **TEK** ]  in combination with the epidermal growth factor EGF like repeats amino acids 1 360 is required for angiopoietin binding.  Ref: 12427764 J Biol Chem, 2003
- Localization of Tie2  [ **TEK** ]  and phospholipase D in endothelial caveolae is involved in angiopoietin 1  [ **ANGPT1** ]  induced MEK ERK phosphorylation and migration in endothelial cells.  Ref: 12890486 Biochem Biophys Res Commun, 2003
- 0.01 but not between VEGF and Ang 1  [ **ANGPT1** ]  or Tie 2  [ **TEK** ]  in human ovarian cancer specimens.  Ref: 12810677 Cancer Res, 2003
- To determine the significance and regulation of the angiopoietin Ang  [ **ANGPT1** ]  pathway in highly vascular human renal cell carcinomas RCCs, this study has investigated the expression of the Ang 1  [ **ANGPT1** ] , Ang  [ **ANGPT1** ]  2, Ang  [ **ANGPT1** ]  4, and Tie2  [ **TEK** ]  genes in a series of normal n = 26 and neoplastic n = 45.  Ref: 12434420 J Pathol, 2002
- To understand the molecular interactions of the angiopoietins with their receptor, we have studied the binding of Ang 1  [ **ANGPT1** ]  and Ang  [ **ANGPT1** ]  2 to the Tie 2  [ **TEK** ]  receptor.  Ref: 12427764 J Biol Chem, 2003
- Furthermore, cellular distribution of Ang1  [ **ANGPT1** ]  and Ang2 mRNA was examined by in situ hybridization, and localization of Tie2  [ **TEK** ]  was studied by immunochemistry.  Ref: 12138242 Oncology, 2002
- The purpose of this study was to evaluate the expression of Tie 2  [ **TEK** ]  and Ang 1  [ **ANGPT1** ]  and 2 in an acetic acid induced gastric ulcer healing process in rats.  Ref: 12768384 J Gastroenterol, 2003
- Western blot analyses showed that HBO enhanced the expression of angiopoietin 2 Ang2 with no effect on the expression of Tie2  [ **TEK** ] , angiopoietin 1  [ **ANGPT1** ] , and VEGF.  Ref: 12176040 Biochem Biophys Res Commun, 2002
- METHODS Since a link has been established in neoplasias between tumor growth and an increased expression of angiogenic growth factors, 136 samples of chorioangiomas and 136 samples of tumor free placental tissue were examined in terms of proliferation rate and expression of the growth factors angiopoietin 1  [ **ANGPT1** ]  and 2, the angiopoietin receptor Tie 2  [ **TEK** ] , PDGF and the PDFG beta receptor.  Ref: 12747234 J Perinat Med, 2003
- However, there was a significant increase in expression of ANG  [ **ANGPT1** ]  2 and its receptor Tie 2  [ **TEK** ]  in menorrhagic tissues, which may be important in destabilizing the endometrial vasculature.  Ref: 12477527 Fertil Steril, 2002
- RESULTS The expression of angiopoietin 1  [ **ANGPT1** ]  messenger RNA and the protein itself and the phosphorylation of TIE2  [ **TEK** ]  were strongly up regulated in the lungs of patients with various forms of pulmonary hypertension, correlating directly with the severity of disease.  Ref: 12571257 N Engl J Med, 2003
- However, using bovine aortic endothelial cells BAEC, we found that only Ang2 expression, but neither Ang1  [ **ANGPT1** ]  nor Tie2  [ **TEK** ] , responded to these two angiogenic stimuli, which was consistent with many previous reports.  Ref: 12686170 Microvasc Res, 2003
- Genetic engineering of precise multimers of the receptor binding domain of angiopoietin 1  [ **ANGPT1** ] , using surrogate multimerization motifs, reveals that tetramers are the minimal size required for activating endothelial Tie2  [ **TEK** ]  receptors.  Ref: 12469114 Nat Struct Biol, 2003
- RESULTS Ang  [ **ANGPT1** ]  2 and Tie 2  [ **TEK** ]  were detected in the endothelial cells of vessels surrounded by tumor cells, occuring early in our study, with immunostaining taking place from day 4 to day 24.  Ref: 12174896 Anticancer Res< MedlineTA>Anticancer Res, 2003
- Introduction of a Tie2  [ **TEK** ]  mutant lacking tyrosine residue 1106 into endothelial cells interferes with Dok R phosphorylation in response to Ang1  [ **ANGPT1** ] .  Ref: 12665569 Mol Cell Biol, 2003
- No significant relationships were observed in clear cell carcinomas between Ang 1  [ **ANGPT1** ] , Ang  [ **ANGPT1** ]  2 and Tie2  [ **TEK** ]  mRNA abundance and patient sex, patient age, or tumour size p > 0.05.  Ref: 12434420 J Pathol, 2002
- In this study, we investigated the expression of Ang 1  [ **ANGPT1** ] , Ang  [ **ANGPT1** ]  2, Tie 2  [ **TEK** ] , and vascular endothelial growth factor VEGF in surgically resected specimens from 46 patients with HCC to determine their potential role in tumor angiogenesis and its progression.  Ref: 12717391 Hepatology, 2003
- METHODS Ang1  [ **ANGPT1** ] , Ang2, and Tie2  [ **TEK** ]  gene expression in 14 normal ovaries with corpus luteum CL and in 19 cases of ovarian cancer were analyzed by polymerase chain reaction of RNA after reverse transcription.  Ref: 12138242 Oncology, 2002
- However, the underlying mechanism for Ang1  [ **ANGPT1** ]  Tie2  [ **TEK** ]  signaling is not fully understood.  Ref: 12890486 Biochem Biophys Res Commun, 2003
- 2 PLD is a new mediator of Ang1  [ **ANGPT1** ]  Tie2  [ **TEK** ]  induced signaling pathway, and it participates in MAPK activation and endothelial cell migration.  Ref: 12890486 Biochem Biophys Res Commun, 2003
- RESULTS The Ang1  [ **ANGPT1** ] , Ang2, and Tie2  [ **TEK** ]  gene expression in normal ovary with CL ranged from 0.18 to 1.06 median 0.5.31 2.64 median 1.01, and 0.10 0.47 median 0.20, respectively.  Ref: 12138242 Oncology, 2002
- These findings suggest that Tie 2  [ **TEK** ]  and Ang 1  [ **ANGPT1** ]  and 2 play an important role in angiogenesis in the early phase of ulcer healing.  Ref: 12768384 J Gastroenterol, 2003
- MATERIAL AND METHODS We have measured the expression of angiogenesis regulating genes angiopoietin 1  [ **ANGPT1** ]  Ang 1  [ **ANGPT1** ] , angiopoietin 1  [ **ANGPT1** ]  Ang  [ **ANGPT1** ]  2 and their receptor Tie 2  [ **TEK** ] , vascular endothelial growth factor VEGF and its receptors VEGFR 1 and VEGFR 2 in sorted population of CD34 and CD34 CD133 cells from human cord blood and bone marrow, and in their differentiating progeny, using real time reverse transcriptase polymerase chain reaction.  Ref: 12605658 Eur J Haematol, 2003
- The study of angiopoietin 1  [ **ANGPT1** ]  Ang 1  [ **ANGPT1** ]  and angiopoietin 2 Ang  [ **ANGPT1** ]  2, ligands of receptor tyrosine kinase 2 Tie 2  [ **TEK** ] , showed that while stroma derived Ang  [ **ANGPT1** ]  2 was increased, epidermal Ang 1  [ **ANGPT1** ]  expression was completely abolished at early papilloma formation.  Ref: 12766907 Mol Carcinog, 2003
- CONCLUSION Our results indicate that there is a difference in the Ang  [ **ANGPT1** ]  Tie2  [ **TEK** ]  gene expression between physiological and pathological angiogenesis in the ovary.  Ref: 12138242 Oncology, 2002
- Expression of Tie 2  [ **TEK** ]  and angiopoietin 1  [ **ANGPT1** ]  and 2 in early phase of ulcer healing.  Ref: 12768384 J Gastroenterol, 2003
- On the other hand, Ang 1  [ **ANGPT1** ]  and Tie 2  [ **TEK** ]  mRNA expression in HCC was not different from that in adjacent liver tissue.  Ref: 12717391 Hepatology, 2003
- Polyclonal antibodies specific for Ang1  [ **ANGPT1** ] , Ang2, Tie2  [ **TEK** ]  and VEGF were used for immunostaining.  Ref: 12815197 Ophthalmic Res, 2003
- To investigate the specific role of Ang2 in the adult vasculature, we generated a nuclease resistant RNA aptamer that binds and inhibits Ang2 but not the related Tie2  [ **TEK** ]  agonist, angiopoietin 1  [ **ANGPT1** ] .  Ref: 12692304 Proc Natl Acad Sci U S A, 2003
- Tie 2  [ **TEK** ]  receptor expression was markedly downregulated in rats treated with MCT, and this was partially restored by gene therapy with Ang 1  [ **ANGPT1** ] .  Ref: 12690034 Circ Res, 2003
- Genetic rescue with Angiopoietin 1  [ **ANGPT1** ]  corrects the lymphatic, but not the angiogenesis, defects, suggesting that Angiopoietin 2 acts as a Tie2  [ **TEK** ]  agonist in the former setting, but as an antagonist in the latter setting.  Ref: 12361603 Dev Cell, 2002
- Thus cyclical mechanical stretch activates the expression of angiopoietin 2 and the Tie2  [ **TEK** ]  receptor, but not angiopoietin 1  [ **ANGPT1** ]  or the Tie1 receptor, in cultured HUVECs.  Ref: 12653688 Clin Sci Lond , 2003
- However, there was significantly greater Ang 1  [ **ANGPT1** ]  p = 0.02, Ang  [ **ANGPT1** ]  2 p = 0.03, and Tie2  [ **TEK** ]  p = 0.04 mRNA abundance in clear cell than in chromophil RCCs.  Ref: 12434420 J Pathol, 2002
- Our purpose was to assess the potential role of the Ang  [ **ANGPT1** ]  Tie2  [ **TEK** ]  system in physiological and pathological angiogenesis in the ovary.  Ref: 12138242 Oncology, 2002
- Immunoprecipitation showed phosphorylation of MPC Tie2  [ **TEK** ]  on tyrosine residues upon stimulation with Ang 1  [ **ANGPT1** ]  or Ang  [ **ANGPT1** ]  2.  Ref: 12876214 J Cell SciJ Cell Sci, 2002
- In contrast, the constitutive expression of Ang1  [ **ANGPT1** ]  and Tie2  [ **TEK** ]  was not affected during hypoxia or deadaptation.  Ref: 12183511 J Appl Physiol, 2002
- RESULTS A higher expression of Ang 1  [ **ANGPT1** ] , Ang  [ **ANGPT1** ]  2, and Tie 2  [ **TEK** ]  mRNAs was detected in CD34 CD133 cord blood cells as compared with CD34 CD133 fraction, but no expression of these genes was detected in burst forming unit erythroid BFU E nor colony forming unit granulocyte macrophage CFU GM colonies.  Ref: 12605658 Eur J Haematol, 2003
- Daily injections 3.75 mg kg sc of DITPA to Sprague Dawley rats affected protein increases in vascular endothelial growth factor VEGF 164, VEGF 188, basic fibroblast growth factor bFGF FGF 2, angiopoietin 1  [ **ANGPT1** ] , and Tie 2  [ **TEK** ]  during the first few days of treatment.  Ref: 12414442 Am J Physiol Heart Circ Physiol, 2003
- Tie2  [ **TEK** ]  gene expression was positively correlated with Ang  [ **ANGPT1** ]  2 expression in cancers p = 0.001 and showed a borderline significant association with Ang 1  [ **ANGPT1** ]  p = 0.06, but there was no significant relationship between Ang 1  [ **ANGPT1** ]  and Ang  [ **ANGPT1** ]  2 p = 0.69.  Ref: 12434420 J Pathol, 2002
- Here, we studied the roles of Ang1  [ **ANGPT1** ] , its natural antagonist Ang2, and their receptor Tie2  [ **TEK** ]  in rat cardiac allograft arteriosclerosis.  Ref: 12628953 Circulation, 2003
- In conclusion, Ang2 and Tie2  [ **TEK** ]  expressions increased after ischemia reperfusion in the rat ventricular myocardium, while Ang1  [ **ANGPT1** ]  and Tie1 expressions did not.  Ref: 12737621 Clin Sci Lond < MedlineTA>Clin Sci Lond , 2003
- mRNAs for VEGF, VEGFR 1 and VEGFR 2, angiopoietin 1  [ **ANGPT1** ] , angiopoietin 2 and their receptor Tie 2  [ **TEK** ]  were localized and quantified by in situ hybridization.  Ref: 12887279 Reproduction, 2003
- In conclusion, our data suggest that both hypoxia and VEGF treatment differentially regulate the angiopoietin Tie2  [ **TEK** ]  system in the two vascular cells and that, particularly in BRP, the regulation of Ang1  [ **ANGPT1** ] , but not Ang2, and Tie2  [ **TEK** ]  expression may play an important role in vascular development.  Ref: 12686170 Microvasc Res, 2003
- In a transwell co culture system, we observed that myeloma cells up regulated the Ang 1  [ **ANGPT1** ]  receptor Tie2  [ **TEK** ]  in human BM endothelial cells.  Ref: 12649156 Blood< MedlineTA>Blood, 2003
- clear cell n = 35, papillary n = 10 human kidney tissues, examined the pattern of Ang  [ **ANGPT1** ]  2 and Tie2  [ **TEK** ]  protein expression, and correlated expression with clinicopathological variables.  Ref: 12434420 J Pathol, 2002
- Conversely, the first 360 amino acids Ig like domain plus EGF like repeats of the Tie 2  [ **TEK** ]  receptor are necessary and sufficient to bind both Ang 1  [ **ANGPT1** ]  and Ang  [ **ANGPT1** ]  2, which suggests that differential receptor binding is not likely to be responsible for the different functions of Ang 1  [ **ANGPT1** ]  and Ang  [ **ANGPT1** ]  2.  Ref: 12427764 J Biol Chem, 2003
- Expression of angiopoietin 1  [ **ANGPT1** ] , angiopoietin 2, and Tie2  [ **TEK** ]  genes in normal ovary with corpus luteum and in ovarian cancer.  Ref: 12138242 Oncology, 2002
- The Tie 2  [ **TEK** ]  receptor tyrosine kinases and ligands, angiopoietin Ang 1  [ **ANGPT1** ]  and 2 have been postulated to play key roles in vascular development.  Ref: 12768384 J Gastroenterol, 2003
- CONCLUSION S Differences in the expression of ANG  [ **ANGPT1** ]  2, Tie 2  [ **TEK** ] , Tie 1, and eNOS were found in menorrhagic endometrium, which may represent a new target for therapeutic intervention to correct menorrhagic conditions.  Ref: 12477527 Fertil Steril, 2002
- The main objective of this study was to identify molecular mechanisms through which angiopoietin 1  [ **ANGPT1** ]  Ang 1  [ **ANGPT1** ] , a ligand for Tie 2  [ **TEK** ]  receptors, influences endothelial cell apoptosis.  Ref: 12074640 Microvasc Res, 2002
- Ang 1  [ **ANGPT1** ] , Ang  [ **ANGPT1** ]  2 and Tie2  [ **TEK** ] , but not Ang  [ **ANGPT1** ]  4 mRNA, were detected in normal and tumour samples.  Ref: 12434420 J Pathol, 2002
- CONCLUSIONS A specific increase of Ang 1  [ **ANGPT1** ]  Ang  [ **ANGPT1** ]  2 ratio in FNH, in the presence of the functional Tie 2  [ **TEK** ]  receptor, might be involved in the formation of hyperplastic and dystrophic vessels of FNH.  Ref: 12612904 Gastroenterology, 2003
- TIE2  [ **TEK** ] , the endothelial specific receptor for angiopoietin 1  [ **ANGPT1** ] .  Ref: 12571257 N Engl J Med, 2003
- Angiopoietins are primarily involved in maturation of both embryonal and adult vasculature, with Angiopoietin 1  [ **ANGPT1** ]  2 being naturally occurring agonists and antagonists of Tie2  [ **TEK** ]  respectively, indicating a very precise level of regulation in vivo.  Ref: 12456344 Front Biosci, 2003
- Expression of Tie 2  [ **TEK** ]  and Ang 1  [ **ANGPT1** ]  and 2 mRNAs was detected in normal gastric tissue and ulcerative tissues by RT PCR.  Ref: 12768384 J Gastroenterol, 2003
- Hypoxia and vascular endothelial growth factor acutely up regulate angiopoietin 1  [ **ANGPT1** ]  and Tie2  [ **TEK** ]  mRNA in bovine retinal pericytes.  Ref: 12686170 Microvasc Res, 2003
- CONCLUSIONS These findings suggest that all forms of pulmonary hypertension are linked by defects in the signaling pathway involving angiopoietin 1  [ **ANGPT1** ] , TIE2  [ **TEK** ] , BMPR1A, and BMPR2 and consequently identify specific molecular targets for therapeutic intervention.  Ref: 12571257 N Engl J Med, 2003
- Angiopoietin 1  [ **ANGPT1** ]  Ang1  [ **ANGPT1** ]  and its receptor, Tie2  [ **TEK** ] , play critical roles in blood vessel formation.  Ref: 12890486 Biochem Biophys Res Commun, 2003
- Angiopoietins are a recently discovered family of angiogenic factors that interact with the endothelial receptor tyrosine kinase Tie2  [ **TEK** ] , either as agonists angiopoietin 1  [ **ANGPT1** ]  or as context dependent agonists antagonists angiopoietin 2.  Ref: 12469114 Nat Struct Biol, 2003
- Angiopoetin 1 Ang1  [ **ANGPT1** ] , angiopoietin 2 Ang2, Tie 1, and Tie 2  [ **TEK** ]  were measured immediately after relief of occlusion, 60 min, 4, and 72 h after reperfusion by Northern blot, Western blot, and immunohistochemical staining.  Ref: 12737621 Clin Sci Lond < MedlineTA>Clin Sci Lond , 2003
